# Assessing the Quality of Reports about Randomized Controlled Trials of Acupuncture Treatment on Mild Cognitive Impairment

**DOI:** 10.1371/journal.pone.0016922

**Published:** 2011-02-25

**Authors:** Xiao Lu, Shang Hongcai, Wang Jiaying, Hu Jing, Xiong Jun

**Affiliations:** 1 Tianjin University of Traditional Chinese Medicine, Tianjin, China; 2 MOE Virtual Research Center of Evidence-Based Medicine, Chengdu, China; 3 Evidence Based Medicine Center in Tianjin, Tianjin, China; 4 Peking University, Beijing, China; 5 Department of Acupuncture, Jiang Xi Hospital of Traditional Chinese Medicine, Jiangxi Province, China; Yale University School of Medicine, United States of America

## Abstract

**Objective:**

To evaluate the reports' qualities which are about randomized controlled trials (RCTs) of acupuncture treatment on Mild Cognitive Impairment (MIC).

**Methods:**

Nine databases including the Cochrane Central Register of Controlled Trials (CENTRAL,2010), PUBMED (1984-5/2010), EMbase (1984-5/2010), MEDLINE (1984-5/2010), CINAL (1984-5/2010), China National Knowledge Infrastructure (CNKI, 1980-5/2010), China Biomedicine Database disc (CBMdisc, 1980-5/2010), VIP (a full text issues database of China, 1989-5/2010) were searched systematically. Hand search for further references was conducted. Language was limited to Chinese and English. We identified 14 RCTs that used acupuncture as an intervention and assessed the quality of these reports against the Consolidated Standards for Reporting of Trials (CONSORT) statement and Standards for Reporting Interventions in Controlled Trials of Acupuncture (STRICTA).

**Results:**

In regard to the items in the CONSORT statement, 13(92.86%) RCTs described baseline demographic and clinical characteristics in each group. 7 (50.0%) mentioned the method of generating the random sequence, only 2 (14.3%) RCTs had adequate allocation concealment. No RCTs used blinding. RCTs reported the sample size calculation. In regard to the items in STRICTA, 10 (71.43%) mentioned the depths of insertion, 6 (42.86%) reported acupuncture response, 11 (78.57%) mentioned the technique of acupuncture, 12 (85.71%) recorded the time, and only 3 (21.43%) RCTs reported the numbers of needles inserted. No RCTs reported the background of the acupuncture practitioners and professional title of practitioners.

**Conclusion:**

The reporting quality of RCTs of acupuncture for mild cognitive impairment was moderate to low. The CONSORT statement and STRICTA should be used to standardize the reporting of RCTs of acupuncture in future.

## Introduction

Mild Cognitive Impairment (mild cognitive disorder) is a clinical state with disease characteristics between normal aging and mild dementia. Most clinical researches show that 44% of the patients diagnosed as cognitive impairment turn into Alzheimer's transit (AD) within 3 years in follow-up studies at an annual average rate of 15% [1]. Recognizing cognitive impairment and identifying individuals at high risk of dementia have become a hot issue in dementia studies in recent years. Lacking in effective drugs and rehabilitation treatment, many patients with mild cognitive impairment begin to try alternative and complementary medicine therapies which include acupuncture treatment. However, despite the publication of a number of case-based research papers the effectiveness of acupuncture on mild cognitive impairment is to be tested and to do so a reliable scientific method is needed. Some research teams decide to adopt the randomized controlled trials (RCTs). RCTs, also known as randomized clinical trials, is a prospective study to test the efficacy and effectiveness of certain treatment or protective measures after comparing the results of the treatment group with those of the control group, thus the subjects involved in the study being randomized divided into two groups [2]. At present, randomized controlled trials (RCTs) is generally considered as the best design plan to verify the efficacy of intervention measures for it boasts rigorous design and scientific methods. It contributes to ensure high-quality systematic reviews, health technology assessment reports, and a variety of other reports for decision analysis, which meets the top requirements of evidence-based medical research [3]Therefore, we have adopted the internationally recognized standards for trial reporting (the CONSORT statement) [4] and the international standards for clinical trials of acupuncture interventions (STRICTA) [5] to evaluate the quality of reports of randomized controlled trials of acupuncture treatment for mild cognitive impairment in order to shed light on its further improvement.

## Methods

### Search strategy

The nine databases we have searched include Cochrane Central Register of Controlled Trials(CENTRAL,2010), PUBMED(1984-5/2010), EMbase(1984-5/2010), MEDLINE(1984-5/2010), CINAL(1984-5/2010), China National Knowledge Infrastructure(CNKI, 1980-5/2010), China Biomedicine Database disc(CBMdisc, 1980-5/2010), and VIP(a full text issues database of China,1989-5/2010). The work was done comprehensively and systematically and further references were searched by hand. Our search is featured by a wide coverage of reports and prospective studies in this field to provide enough information for research.

However, our search confined itself to reports written or published either in Chinese or in English. For one thing, it is because a majority of journal papers on acupuncture are published in these two languages and it is apparently impossible for reports written in Chinese to be included in non-Chinese databases. Besides, our team members were unable to read reports in languages other than Chinese and English. It may constitute to a limitation of this study.

We did not limit our search to a specific publication date range at the beginning of this study because we wanted to find as many reports as possible concerning mild cognitive impairment in the above databases. However, the databases themselves have such limitations.

### Eligibility of the study

We included original reports in Chinese or English that described acupuncture as an intervention, stated the method, and provided specific data in this study and excluded commercials, speeches, letters and meeting reports.

### Selection of reports to be studied

**Figure 1:**
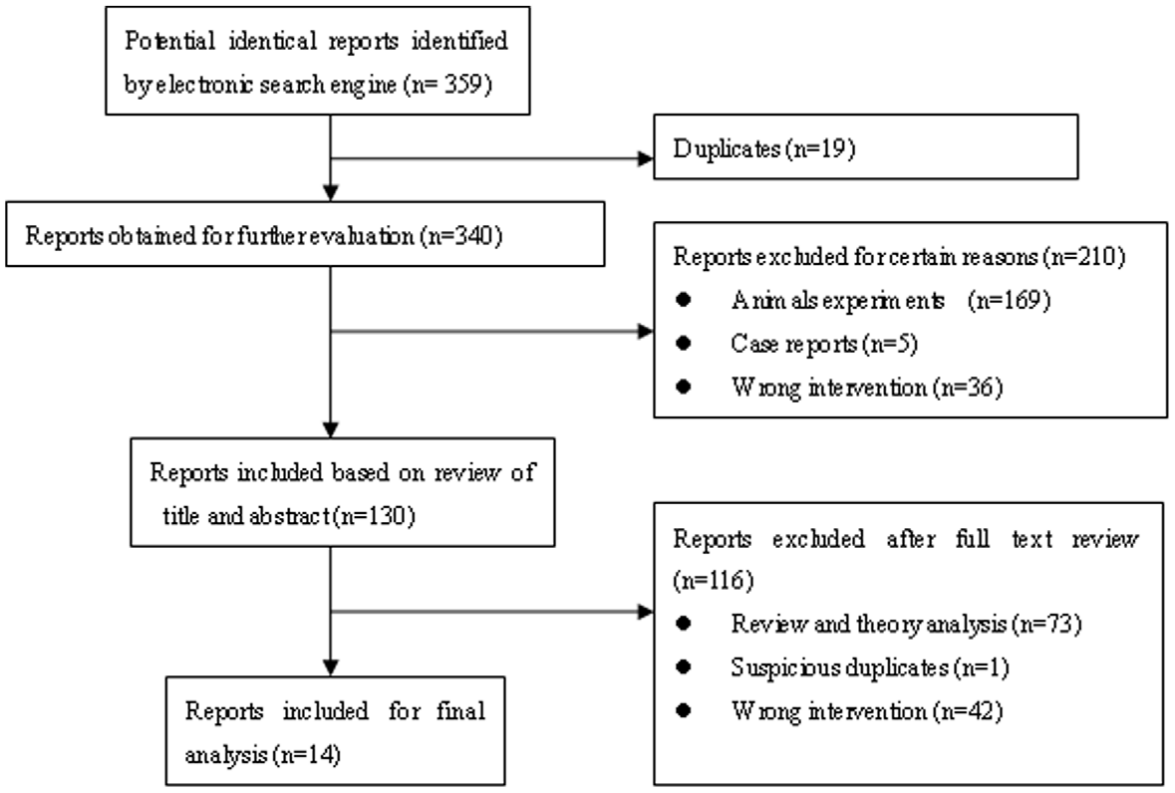
Flow chart of reports selection. This figure shows the process of the selection. The researchers applied the search method to find 359 reports related to the topic. One researcher (XL) picked out 19 duplications of these reports by the software NoteExpress and 169 reports of animals experiments, 5 cases and 36 reports of the wrong intervention by scanning the title and abstract of the citation retrieved by the selection search engine (first scanning). Another researcher (WJY) then viewed the full text of all potentially eligible reports obtained and picked out 73 reviews and theory analyses, one suspicious duplicate and 42 reports of the wrong intervention. At last, 14 reports are included for final analysis.

At first, one researcher (XL) picked out duplications of these reports using NoteExpress, and scanned the title and abstract of the citation retrieved by the selection search engine (first scanning). Another researcher (WJY) then viewed the full text of all potentially eligible reports obtained. After that, similar reports were marked “suspicious duplications” and compared. If the similarity rate of these articles was beyond 80% (including 80%), the one with the fullest information was included while the others were omitted. Disagreements between the two researchers were discussed by the whole team and were solved at last. (Figure 1)

### Data abstraction

Two researchers (XL and WJY) compiled a table to list the general information of the final included reports. Items of the table include report title, journal name, year of publication, type of control, number of participants and country of publication. Any missed information was coded as “not reported” and disagreements were resolved after getting more information from related references before finalizing the table. Two reviewers scanned the full text of all 14 reports and filled the table.

### Assessment methods

We assessed the quality of these reports by CONSORT and STRICTA standards. CONSORT (consolidated standards of reporting trails) was introduced in 1996 [6] in an attempt to alleviate the problems arising from inadequate reporting, and it was revised five years later [7]. STRICTA (standards for reporting interventions in controlled trials of acupuncture) was compiled between late 2001 and early 2002 [8].

The CONSORT statement comprises a 22-item checklist including abstract, background, participants, interventions, objectives, outcome measures, sample size, sequence generation, allocation concealment, implementation, blinding, statistical methods, participant flow, recruitment, demographic data, numbers analyzed, outcomes and estimation, ancillary analyses, adverse events, interpretation and overall evidence.

STRICTA consists of 15 items including acupuncture rationale, points used, number of needle inserted depths of insertion, response elicited, needle stimulation, needle retention time, needle type, treatment regimen, co-interventions, practitioner background, duration of training, length of clinical experience, expertise, control interventions. The above information can also be found in Table 2 and Table 3.

Two researchers were required to study the CONSORT and STRICTA standards before making assessment to ensure correct understanding of every item listed in the two checklists. If they had any difficulties or disagreements in the process, they solved the problem in discussions. The 14 reports were then assessed according to the two standards respectively and each item was marked “M” (mentioned) or “NM” (not mentioned).

## Results

### Report selection

A total of 359 potentially relevant reports were identified from the databases for review. 229 publications were considered to be unfit for our study after a preliminary review of title and abstract. Another 74 were excluded after full-text review. At last a total of 14 full-text reports [9]–[22] were regarded eligible for a more systematic analysis.

### Characteristics of the Reports

**Figure 2:**
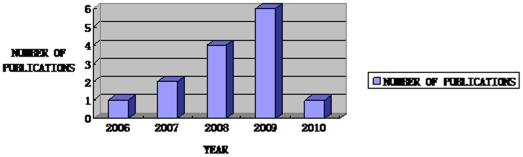
Number of reports published in the recent five years. According to the Table 1, the years of publication of included reports is mainly during these five years, namely from the 2006 to 2010. Figure 2 shows one report is published in 2006, two in 2007, four in 2008, six in 2009, and one in 2010.

The characteristics of all the included reports are presented in Table 1. All the 14 reports were found in 11 different medical journals published by six different publishing houses. Sample sizes ranged from a small pilot study of 17 subjects to a large scale, and the largest sample included 104 subjects. Only one report was published in America. All the 14 studies included in our assessment were published during the year 2006–2010, while in 2009 the number of reports published reached the peak (43%) (Figure 2).

**Table 1:** Characteristics of the reports included (N = 14).

Report title	Journal name	Year of publication	Type of control	Number of participants	Country of publication
He 2008	JOURNAL OF PRACTICAL TRADITIONAL CHINESE MEDICINE	2008	aceglutamide	60	China
Yu 2007	SHAANXI JOURNAL OF TRADITIONAL CHINESE MEDICINE	2007	Nimodipine	67	China
Sun 2009	JOURNAL OF CLINICAL ACUPUNTURE AND MOXIBUSTION	2009	duxil tablet	60	China
Hou 2010	WORLD JOURNAL OF INTEGRATED TRADITIONAL AND WESTERN MEDICINE	2010	Sham acupuncture	36	China
Hou 2009	JOURNAL OF BEI JING UNIVERSITY OF TRADITONAL CHINESE MEDICINE	2009	Sham acupuncture	40	China
Sun 2007	Chinese Journal of Traditional Medical Science and Technology	2007	Aricept	62	China
Su 2006	LIAO NING JOURNAL OF TRADITIONAL CHINESE MEDICINE	2006	Nimodipine	100	China
Zhou 2008	SHANGHAI JOURNAL OF ACUPUNTURE AND MOXIBUSTION	2008	Sham acupuncture	60	China
Zhou 2008	GUANGXI JOURNAL OF TRADITIONAL CHINESE MEDICINE	2008	Regular treatment	60	China
Liu 2009	SHANGHAI JOURNAL OF ACUPUNTURE AND MOXIBUSTION	2009	Aricept	17	China
Chen 2008	JOURNAL OF EMERGENCY IN TRADITIONAL CHINESE MEDICINE	2008	Regular treatment	60	China
Chen 2009	Chinese Journal of Traditional Medical Science and Technology	2009	Regular treatment	60	China
Feng 2009	Jilin Journal of Traditional Chinese Medicine	2009	Sham acupuncture	104	China
Pei 2009	THE JOURNAL OF ALTERNATIVE AND COMPLEMENTARY MEDICINE	2009	Conventional rehabilitation	38	America

### Use of CONSORT and STRICTA

At first we found that none of the reports included in the assessment explicitly mentioned the CONSORT or STRICTA statement. After referring to the official websites of the 11 journals, we found that only two journals require manuscripts to be submitted to meet the CONSORT or STRICTA standards. However, we found out later that there were 3 reports that included 19 items of CONSORT and 12 items of STRICTA checklist, which means that the two statements have actually been employed by more authors.

### Reporting Assessment Using CONSORT and STRICTA

The results are presented in Table 2 and Table 3. Not a single report fulfilled either all the 21 items of the CONSORT checklist or the 15 STRICTA items. Above all, the main problem we found was a lack of information concerning method of blinding, practitioner background, and participant flow and recruitment.

**Table 2:** Reporting quality of 14 RCTs based on CONSORT.

Total number of items	Number of reported RCTs (%)	
Abstract	7	50.00
Introduction		
Background	11	78.57
Methods		
Participants	12	85.71
Interventions	13	92.86
Objectives	12	85.71
Outcomes	13	92.86
Sample size	14	100.00
Randomization		
Sequence generation	7	50.00
Allocation concealment	2	14.29
Implementation	1	7.14
Blinding	0	0.00
Statistical methods	12	85.71
Results		
Participant flow	0	0.00
Recruitment	0	0.00
Baseline data	13	92.86
Numbers analyzed	12	85.71
Outcomes and estimation	10	71.43
Ancillary analyses	4	28.57
Adverse events	3	21.43
Discussion		
Interpretation	13	92.86
Overall evidence	7	50.00

**Table 3:** Reporting quality of interventions in 14 RCTs based on STRICTA.

Intervention	Number of reported RCTs (%)	
Acupuncture rationale	13	92.86
Needling details		
Points used	14	100
Number of needles inserted	3	21.43
Depths of insertion	10	71.43
Response elicited	6	42.86
Needle stimulation	11	78.57
Needle retention time	12	85.71
Needle type	7	50.00
Treatment regimen	11	78.57
Co-interventions	3	21.43
Practitioner background	0	0.00
Duration of training	0	0.00
Length of clinical experience	0	0.00
Expertise	0	0.00
Control intervention(s)	11	78.57

#### CONSORT

Seven CONSORT items were mentioned in less than 30% of the total reports. They are methods of blinding, participant flow and recruitment (0%), implementation of randomization (7.14%), description of allocation concealment (14.29%), adverse events of acupuncture (21.43%) and outcomes of ancillary analyses (28.57%).

Another four CONSORT items were included in more than half of all the reports, i.e., abstract, sequence generation and overall evidence in the section of discussion.

#### STRICTA

None of these studies gave any information about practitioner background which was made up of expertise, duration of training and length of clinical experience. Two STRICTA items, namely, number of needles inserted and co-interventions were explained in 21.43% of the reports. Another two items were made clear in less than half of the reports, i.e., needle type (50%) and response elicited.

## Discussion

### Summary of the study

The main strength of our study is that we have searched a total of nine databases in order to encompass all the related reports. Moreover, reports selection, data collection, reports assessment and all the other work were carefully conducted by well-trained researchers.

Another important strength of our study is that we use the CONSORT and STRICTA statement to evaluate RCTs of acupuncture. Both of them are the most widely accepted criteria for reporting quality assessment in acupuncture studies. Plain in words and ready for use, the two statements were developed in the aim of alleviating the problems arising from inadequate reporting of randomized controlled trials. They are powerful tools, as has been proved by the fact that the majority of their items were well reported, with a few exceptions (7 CONSORT items and 9 STRICTA ones were poorly reported).

We believe it is high time to call for clinical researchers to follow the two statements in conducting RCTs and in thesis writing. At present, Chinese journals set down no requirements for the author to follow the CONSORT or STRICTA statement in reporting Therefore, we recommend that mainstream Chinese journals make the application of CONSORT or STRCITA a prerequisite, so that transparency and integrity of RCTs can be ensured. More importantly, for policy makers, it is urgent to let more clinical researchers know about the CONSORT and STRICTA statement.

### Limitations

Although we have assessed the 14 reports comprehensively and systematically, there are still some limitations. First, the reports included in our study were published exclusively in years between 2006 and 2010 although we didn't make any limitations on the date of publication during sample selection. The fact that the selected reports cover a narrow time span may harm the value of our study. However, it is worth mentioning that the whole concept of mild cognitive impairment was not put forward until 1999 by American scholar Petersen. Apparently it took some time for the diagnostic criteria to be introduced to China and to be widely accepted by Chinese scholars. In addition, it probably cost more time for acupuncture, a complementary and alternative intervention, to be involved in the treatment. Recently, some research groups propose the need to test the efficacy of acupuncture treatment after three or four years' clinical practice in cognitive impairment patients, thus RCTs were conducted. Nevertheless, at the beginning we didn't realize it was impossible for us to find any target report before 1999 if sample selection was conducted with “mild cognitive impairment” as a search word, which means we have done something useless and our search strategy remains to be improved.

According to resources and databases accessible to us, the earliest RCTs were carried out in 2006. However, there might be earlier articles in the database that we didn't find for certain reasons, such as language competence. We limited our search to reports written in Chinese and English, but the English report turned out to be one in number. It seems that our quality review was confined to domestic reports. Results may vary if we were able to include more reports in other languages, but this requires our team members to attain a higher level of language skills, a target we are confident to reach in the future.

Another limitation lies in that we didn't follow the rule of blinding when assessing and allocating the reports. Researches who did the scanning were directly involved in the evaluation process. This is because we believe two researchers are enough to evaluate the whole 14 selected reports and allocation was unnecessary. However, although the two researchers had studied the same version of the checklist items beforehand, they came out with different opinions on certain issues. This shows that assessment bias still exists.

### Conclusion

Our study suggests that the reporting quality of recently published reports of RCTs of acupuncture treatment for mild cognitive impairment remains to be improved. However, we have found only one published report had formally appealed for the adoption of CONSORT in future research. With more articles published on this subject, it is necessary to call for the wide application of the CONSORT and STRICTA standards to improve the quality of reporting, and this study should be pursued further with a greater number of reports on this subject assessed and analyzed.
